# The Panel Doctor

**Published:** 1921-09-24

**Authors:** 


### Letters to a Nurse.

### II.—THE PANEL DOCTOR.

Dear Barbara,—So you have learned to be scornful of the Panel Doctor, have you? "All very well for the servants—but . . . !" My dear—cut it out. Once I knew a doctor who refused to attend servants. He would answer the call of no one lower than an employer. He is, deservedly, dead. He died rather painfully. Snobbishness is bad everywhere. It is the unforgivable sin in a doctor.

People who are scornful of the Panel Doctor, *qua* Panel Doctor, deserve to be bereft of any doctor at all in their hour of need. For, and this is fundamentally true, the only difference between an honest Panel Doctor and any other variety of honest doctor is this—one accepts service and pay from the Government; the other does not. One is, to some extent, a public servant; the other is purely a private adventurer.

I am a Panel Doctor. I have been a Panel Doctor from the beginning, and I assert cheerfully that my Panel work gives me more satisfaction than any other work I do. "Why?" you ask. Just because the financial, the business, the fee-getting element is eliminated in Panel work. I am paid a certain sum per annum for each person on my list. I give to them just what service each one of them requires. No one can suggest that I am trying to run up a bill; no one can hint that I am putting in unnecessary attendances. I do what I think I ought to do, and if people consider that I fall short of what I ought to do they can complain. There is a special sub-committee whose business it is to hear complaints. I have been hauled up before it once—on a charge of refusing to give a prescription to a patient whom I had not seen! I left the Court without any stain on my character.

Forget the libels on the Panel Doctor. He is just as competent and just as useful (or the reverse) as the Harley Street man. He costs less, but he is none the worse for that.

Of course, there are Panel Doctors and Panel Doctors. But so there are Harley Street men and Harley Street men. A first-class Panel Doctor is about as good as anything Harley Street has to offer; and an indifferent, or careless, Panel Doctor is, perhaps, rather worse than anything you can find in Harley Street. But the average is about the same—Panel Doctor or Harley Street man.

Don't forget—the *good* Panel Doctor is often a G.P. of experience. He was in practice many years before the Insurance Act came into being, and, if he is any use at all, he employed his ears and his eyes and his general intelligence to good purpose during those years. A sound G.P. can give points to Harley Street in the matter of diagnosis; and, as to treatment, he just leaves Harley Street guessing.

Have you ever heard of Sir James . . . ? My word! I almost dragged in a personality there! Any way, I'll bet you never did hear of him. He was a G.P. and he has done more for medicine than anyone else living.

Cut out your scorn, my dear—cut it out.

Your affectionate

Uncle Luke.

